# Temporal sampling helps unravel the genetic structure of naturally occurring populations of a phytoparasitic nematode. 1. Insights from the estimation of effective population sizes

**DOI:** 10.1111/eva.12352

**Published:** 2016-02-11

**Authors:** Pierre‐Loup Jan, Cécile Gracianne, Sylvain Fournet, Eric Olivier, Jean‐François Arnaud, Catherine Porte, Sylvie Bardou‐Valette, Marie‐Christine Denis, Eric J. Petit

**Affiliations:** ^1^ INRAUMR1349 IGEPPF‐35653Le Rheu CedexFrance; ^2^ INRAUMR985 ESEF‐35042Rennes CedexFrance; ^3^ UMR CNRS 8198 Évolution, Écologie et PaléontologieUniversité Lille 1 – Sciences et Technologies59655Villeneuve d'Ascq CedexFrance

**Keywords:** *Beta vulgaris* spp. *maritima*, effective population size, *Heterodera schachtii*, temporal sampling, wild nematode populations

## Abstract

The sustainability of modern agriculture relies on strategies that can control the ability of pathogens to overcome chemicals or genetic resistances through natural selection. This evolutionary potential, which depends partly on effective population size (*N*_*e*_), is greatly influenced by human activities. In this context, wild pathogen populations can provide valuable information for assessing the long‐term risk associated with crop pests. In this study, we estimated the effective population size of the beet cyst nematode, *Heterodera schachtii*, by sampling 34 populations infecting the sea beet *Beta vulgaris* spp. *maritima* twice within a one‐year period. Only 20 populations produced enough generations to analyze the variation in allele frequencies, with the remaining populations showing a high mortality rate of the host plant after only 1 year. The 20 analyzed populations showed surprisingly low effective population sizes, with most having *N*_*e*_ close to 85 individuals. We attribute these low values to the variation in population size through time, systematic inbreeding, and unbalanced sex‐ratios. Our results suggest that *H. schachtii* has low evolutionary potential in natural environments. Pest control strategies in which populations on crops mimic wild populations may help prevent parasite adaptation to host resistance.

## Introduction

Modern agriculture requires the development of integrated strategies that can control epidemics with reduced pesticide use (Pimentel 2005; Berny 2007; Meissle et al. 2010). These strategies involve reconciling agronomic, economic, and sociological constraints with biological realities. For example, the use of chemicals or genetic resistance, which are commonly employed for parasite control, may not be sustainable if the parasite possesses the evolutionary potential to overcome these resistance mechanisms via natural selection (see Mundt 2014 for a recent review and Fournet et al. 2013 for an example based on a cyst nematode). Thus, one particular challenge ahead is to build strategies that account for this evolutionary potential, which is a determining factor of long‐term pathogen‐related risks (McDonald and Linde 2002; Barrett et al. 2008). The evolutionary potential, which is the ability for a population to adapt and evolve in its environment, is a population genetic concept that helps estimate the probability that a pathogen will overcome management strategies such as those relying on genetic resistance. The evolutionary potential depends on mutation and gene flow, effects of which are proportional to the product of their intrinsic rates and effective population size, genetic drift, which is inversely related to effective population size, and selection, which outcomes depend both on selection differential and effective population size (McDonald and Linde 2002; see also box 5.1 in Frankham and Kingsolver 2004 for an example of a mathematical model of evolutionary potential). The effective population size (*N*_*e*_) is a key evolutionary parameter that corresponds to the size of an idealized population (balanced sex ratio, random mating, Poisson distribution of family size, nonoverlapping generations, etc.) subjected to the same amount of genetic drift as the population of interest. Effective population size thus informs about the strength of genetic drift within populations and depends on demographic factors such as census population size, dispersal ability, or the mating system. It is considered as a fundamental factor in the estimation of the risk associated with resistance in parasite populations (McDonald and Linde 2002; Barrett et al. 2008; Charlesworth 2009). Small effective population sizes imply that genetic drift predominates, making populations less prone to respond to natural selection. Thus, populations with small effective sizes are likely to have reduced capacity for adaptation and, in the special case of pathogens, a low probability of overcoming chemical controls or host resistance.

Crop pathogens or parasites can also be found in wild hosts (Barrès et al. 2008; Barrett et al. 2009; Gracianne et al. 2014), particularly because wild hosts are the initial host species before domestication or because parasites can also subsequently shift to other cultivated plants (Stukenbrock and McDonald 2008). Once in a cultivated environment, the geographical distribution, host associations, and disease dynamics of pests are mainly influenced by human activities (Morgan et al. 2012; Bousset and Chèvre 2013). Knowledge of the genetic structure of parasite populations in natural areas free of human influence is thus not only a valuable approach for understanding the evolutionary histories of these populations, but also is important for calibrating the evolutionary potential of populations. The phylum Nematoda contains more than 4100 plant‐parasitic species, causing damages estimated at US $80 billion per year (Hugot et al. 2001; Jones et al. 2013). Nematode management strategies are focused mainly on crop rotation and the use of genetically resistant crops (Van der Putten et al. 2006; Nicol et al. 2009), and it has already been recognized that the control of plant‐parasitic nematodes would greatly benefit from knowledge on spatial and evolutionary patterns that arise in wild ecosystems (Van der Putten et al. 2006). Nonetheless, to date, no studies have investigated the effective population sizes of these parasites. We thus set out to assess the effective population size in a plant‐parasitic nematode, *Heterodera schachtii*. This cyst nematode infects the roots of the sugar beet (*Beta vulgaris* spp. *vulgaris*) and is one of the most damaging pathogens of sugar beet crops (Zhang et al. 2008). Crop rotation and intercropping decrease nematode populations, but they are not compatible with intensive agricultural practices (Van der Putten et al. 2006; Curto 2008). Genetically resistant sugar beet is thus considered as the most promising nematode management method. However, the development of resistance‐breaking strains of this nematode is a main concern (Zhang et al. 2008; Thurau et al. 2011). Although resistance to *H. schachtii* has not yet been defeated in commercial crops, we know that natural populations can be virulent against specific resistance genes (Müller 1998). Furthermore, adaptation to nematode resistance has already been observed on populations of potato cyst nematodes. *Globodera pallida* has adapted to resistant varieties in laboratory experiments after only 5–8 generations, and a sister species, *Globodera rostochiensis*, has rapidly overcome the resistance gene H1 in fields in the Netherlands (Zaheer et al. 1993; Fournet et al. 2013).

Under field conditions, the population genetic structure of cyst nematodes is thought to be heavily influenced by human activities (Alenda et al. 2014). The wild sea beet (*Beta vulgaris* spp. *maritima*), the wild ancestor of all cultivated beets, also is one of the hosts of *H. schachtii* in natural ecosystems (Maas and Heijbroek 1982). Our study focused on wild populations of *H. schachtii* to assess effective population sizes of wild populations free of human‐related disturbance.

There are a wealth of methods for estimating contemporary effective population size (Wang 2005; Palstra and Ruzzante 2008; Luikart et al. 2010). They can be divided into two basic categories. First, single‐sample estimators extrapolate *N*_*e*_ from parameters such as linkage disequilibrium and heterozygote excess, or summary statistics included in an approximate Bayesian computation (Pudovkin et al. 1996; Tallmon et al. 2008; Waples and Do 2008). Second, temporal methods consider the variation in allele frequencies between temporally spaced samples as the impact of genetic drift, which can be translated into *N*_*e*_ estimates. Both approaches have their advantages and drawbacks, but the biological characteristics of *H. schachtii* may especially affect the results of single‐sample estimators, namely the potentiality for inbreeding and fine‐scaled substructuring of populations (Plantard and Porte 2004; Montarry et al. 2015). Systematic inbreeding prevents the extrapolation of effective size from the excess of heterozygotes (Zhdanova and Pudovkin 2008) or linkage disequilibrium (Waples and Do 2010), and population substructuring is known to downwardly bias single‐sample estimators (Holleley et al. 2014). Therefore, to avoid these potential biases, we estimated *N*_*e*_ of *H. schachtii* populations using temporal methods.

To estimate the effective size of wild populations of *H. schachtii* using temporal methods, we sampled 34 populations parasitizing wild sea beets in Normandy, France, twice, with a sampling interval of 1 year. Beyond the basic understanding of genetic structure, the *N*_*e*_ of these populations may give insights into the understanding of the evolutionary dynamics of *H. schachtii* and help assess the long‐term risk that host resistance will be overcome in the field.

## Materials and methods

### Biology of *Heterodera schachtii*

*Heterodera schachtii* is a cyst nematode that infests the roots of wild sea beets and cultivated sugar beets. Active dispersal abilities of *H. schachtii* are considered to be extremely limited—about a few centimeters—due to their small size and their inability to move in anything but fluids (Plantard and Porte 2004). Thus, one nematode population is defined at the scale of a single, individual plant of *B. vulgaris* spp. *maritima*. Cyst nematodes are characterized by the solidification of the female cuticle after mating with one or several males (Triantaphyllou and Esbenshade 1990). Females then become a cyst that contains the eggs of the next generation. Eggs can be dormant for a long time, especially when cysts do not receive the proper stimuli, and hatching time can be extremely variable among cysts (Zheng and Ferris 1991). The development of larvae involves distinct stages, with the second‐stage juvenile (J2) being the only larval stage that is free‐living and mobile. The genetic diversity and population structure of *H. schachtii* has been studied in sugar beet fields and substantial departures from Hardy–Weinberg (HW) expectations suggest extensive inbreeding due to mating between relatives (Plantard and Porte 2004). A recent study conducted both on field and wild populations suggested that HW departures also may be explained by genetic substructuring due to Wahlund effects at the scale of the host plant (Montarry et al. 2015).

### Biological material and sampling design

**Figure 1:**
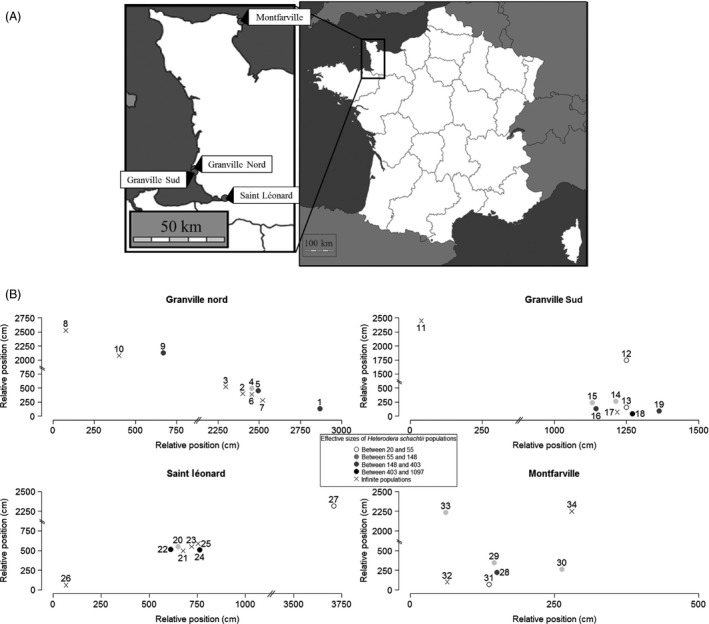
(A) Beach locations and (B) relative locations of sampled populations in each beach. Symbols show effective sizes estimated with the pseudo‐likelihood method (see text). Effective population size classes (and the corresponding gray scale) were taken from Fig. 2.

Soil samples were collected around roots of *B. vulgaris* spp. *maritima* plants on four different beaches in Normandy, France. The beaches, namely Montfarville (1°14′31″ W, 49°38′46″ N), Granville Nord (1°34′10″ W, 48°48′22″ N), Granville Sud (1°34′5″ W, 48°48′12″ N), and Saint Léonard (1°26′42″ W, 48°39′17″ N), were separated by distances ranging from 300 m to 150 km (Fig. 1), a geographic range that is similar in extent to the geographic range that was sampled in previous investigations on this nematode conducted in sugar beet fields (Plantard and Porte 2004). Populations of the host plant, the sea beet, are often composed of individuals clustered in geographically and genetically distinct patches (De Cauwer et al. 2010). Thirty‐four sea beet plants, with a maximum of 10 plants on each beach, were sampled. The temporal method requires two distinct sampling sessions, ideally separated by at least five generations (Waples and Yokota 2007). In the first sampling (November 2012), the sampled plants were marked with numbered plastic tags for easy identification during the second sampling session (November 2013). We recorded whether plants were dead or alive at the second sampling session. Cysts of *H. schachtii* were extracted from soil samples using homemade sieves (250 μm and 800 μm) and manual examination of filtrates. Cysts were stored at 4°C in moistened sand before molecular characterization.

### Molecular characterization and genotyping

For each sampled nematode population, 40 second‐stage juvenile larvae were used to perform DNA extraction and each larva was extracted from a different and randomly chosen cyst, to avoid family structure biases caused by sibling relationships. A soil sample can contain cysts from different *Heterodera* species; therefore, molecular characterization based on the restriction profiles of ITS sequence was used for species identification. By multiplying the ratio of *H. schachtii* among the 40 larvae with the number of cysts we sampled, we estimated the number of *H. schachtii* cysts contained in our samples. DNA extraction, PCR amplification of ITS sequence, and digestion of PCR products were performed as described in Gracianne et al. (2014).

We ultimately genotyped 761 and 854 *H. schachtii* individuals in 2012 and 2013, respectively, using eight microsatellite loci, named Hs33, Hs36, Hs55, Hs56, Hs68, Hs84, Hs111, and Hs114 and described in Montarry et al. (2015). Microsatellites PCR products were analyzed on an ABI Prism^®^ 3130xl sequencer (Applied Biosystems^™^, ThermoFisher Scientific, Waltham, MA, USA), and allele sizes were identified using the automatic calling and binning procedure of GeneMapper v4.1 (Applied Biosystems^™^), with any irregular results evaluated manually. Samples with dubious genotypes were reamplified.

### Characterization of basic genetic parameters

Single and multilocus departures from Hardy–Weinberg (HW) equilibrium were tested for all populations on each beach using permutation tests (10 000 permutations) adjusted for multiple tests with Bonferroni corrections, as implemented in the software Fstat version 2.9.3 (Goudet 1995). This procedure is based on the estimation of *F*_IS_, and its statistical significance, for each population. The genetic diversity of nematode populations was evaluated by estimating expected heterozygosity (H_e_) and allelic richness (Ar) using Fstat. Allelic richness was estimated using the rarefaction method described in El Mousadik and Petit (1996).

### Number of generations produced between the two sampling sessions

The temporal method requires knowledge of the number of generations produced between sampling periods. To date, there are no data on the generation time of *H. schachtii* in wild populations. However, *H. schachtii* produces about four generations a year in cultivated fields (Subbotin et al. 2010). Sugar beets can be parasitized over approximately 7 months in cultivated fields before being harvested in autumn, whereas sea beets are perennial plants in our surveyed geographical areas (Hautekèete et al. 2002; De Cauwer et al. 2010, 2012). Thus, we assumed that wild populations of *H. schachtii* produce at least four generations in 1 year and considered this estimate to be the minimum number of generations produced per year. The generation time of cyst nematodes also strongly depends on temperature (Griffin 1988), as measured by Kakaire et al. (2012) under controlled laboratory conditions. We modeled their results on minimum generation time at different temperatures with a nonlinear regression model based on the least squares method and a Gaussian curve, which visually fit the data point provided by Kakaire et al. (Fig. S1), using the software R (R Core Team 2014, version 2.12.2). This model was then used along with monthly temperature data provided by two weather stations (Météo‐France data), one located near Granville and Saint Léonard, and the other one located near Montfarville, to estimate the maximum number of generations produced by *H. schachtii* between the two sampling sessions. In subsequent analyses, we used these minimum and maximum numbers of generations as well as their median value to evaluate the impact of this demographic parameter on effective population size estimations.

### Estimating effective population size

Two methods were applied to estimate the effective population sizes of *H. schachtii*. We used the pseudo‐likelihood method of Wang (2001) implemented in the software MLNE 1.0 (Wang and Whitlock 2003). This method has been demonstrated to be the most reliable among 14 estimation methods, including seven temporal methods (Gilbert and Whitlock 2015). We also used the moment‐based method developed by Jorde and Ryman (2007) and implemented in the software NeEstimator v2 (Do et al. 2014). Alleles with frequencies below 0.05 were excluded to avoid any bias caused by rare alleles, as described in Do et al. (2014). Both methods are complementary: the moment‐based method yields unbiased but rather imprecise *N*_*e*_ estimates, while the pseudo‐likelihood method gives *N*_*e*_ estimates that can be slightly upwardly biased, but that are more precise (Jorde and Ryman 2007; Do et al. 2014). If the variation in allele frequencies between temporal samples is too low compared with sampling error, the moment‐based method yields an infinite estimate of effective population size. Given differences in computing techniques, the pseudo‐likelihood estimate in such cases reaches the upper limit of possible values (here set to 35 000 individuals), which is then considered infinite as well.

To check whether the two kinds of *N*_*e*_ estimators gave similar and biologically meaningful results, the two set of results were compared for significant differences using a Wilcoxon signed‐rank test on populations characterized by ‘finite’ effective population size results.

Both methods assume neither migration nor selection and that the variation in allele frequencies between temporal samples is due only to genetic drift and sampling errors. To verify this hypothesis, we performed an exact test of homogeneity in allele frequencies between temporal samples to detect significant changes in allele frequencies using the software Genepop v4.2 (Raymond and Rousset 1995). Populations with significant changes were then selected as candidates to test for the presence of nonrandom variation in allele frequencies (which would be caused by migration or selection) using the generalized test described in Waples (1989). The generalized test was performed *a posteriori* because it requires an estimation of effective population size. To this end, we used the pseudo‐likelihood estimation of *N*_*e*_, thought to be more accurate, assuming the median number of generations (see below) between the temporal samples to perform this test.

Waples and Wilcoxon tests were computed with R (R Core Team 2014, version 2.12.2).

## Results

### Number of generations produced between the two sampling sessions

The modeled relationship between temperature and the minimum generation time of *H. schachtii* is shown in the Figure S1. The resulting maximum number of generations produced each month ranged from 0.2 to 1.2 (Fig. S2). Overall, adding up all of the generations over the entire year resulted in 9.9 generations for Montfarville beach and 10.6 for the three other surveyed beaches. Wild *H. schachtii* populations may thus have produced up to 10 generations between the two sampling sessions. The number of generations that elapsed between the two sampling sessions was therefore set to the minimum (four generations), maximum (10 generations) and their median values (seven generations) in subsequent analyses.

### Estimations of effective population sizes

**Figure 2:**
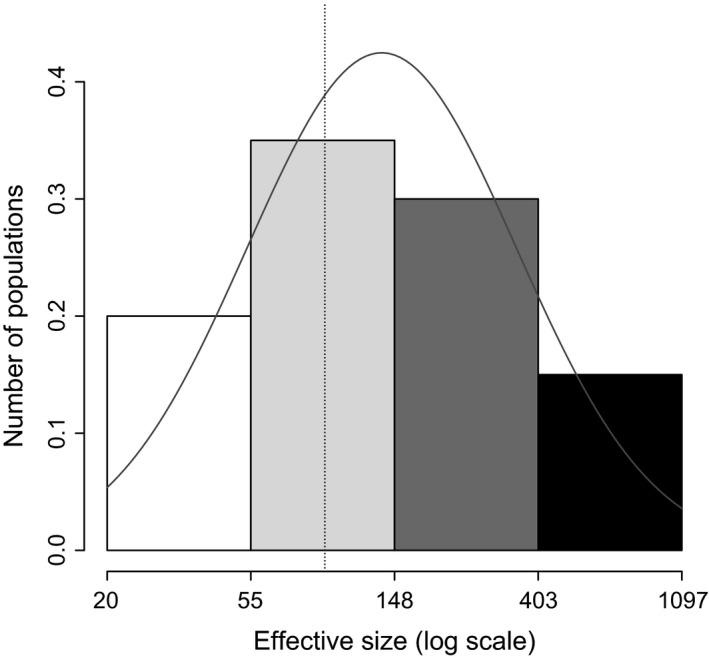
Effective sizes of finite populations of *Heterodera schachtii* (log scale). Effective sizes were estimated with the pseudo‐likelihood method (see text). The dashed line indicates the mode of this distribution. The gray curve corresponds to a fitted log‐normal distribution.

**Figure 3:**
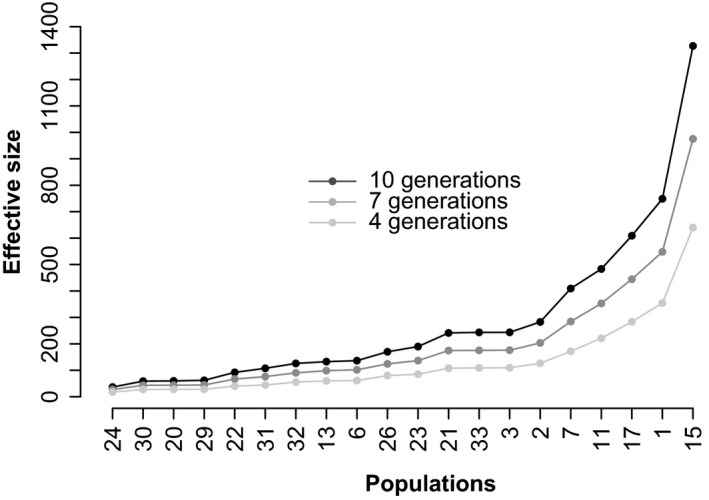
Influence of the number of generations on effective sizes of finite population of *Heterodera schachtii*. Effective sizes were estimated with the pseudo‐likelihood method (see text). Populations are ordered from the lowest to the greatest effective size.

Characterization of the microsatellite loci and populations used for effective population size estimation are presented in Tables 1 and 2, respectively. When considering the median value of seven generations, effective population sizes were extremely variable among populations, with 14 populations showing an infinite effective population size for at least one estimator (Table 3). The other 20 populations showed finite effective population size estimates with both methods. These populations will be called ‘finite populations’ as opposed to the 14 remaining ‘infinite populations’. Based on a seven‐generation span among temporal samples, mean effective sizes of finite populations were equal to 218 and 209 individuals for the moment‐based and the pseudo‐likelihood estimates, respectively. The distribution of the effective size of finite populations fits a log‐normal distribution (Fig. 2), and the modes of these distributions were 80 for the moment‐based estimator and 91 for the pseudo‐likelihood estimator. As expected (Jorde and Ryman 2007), the moment‐based method had greater variance (80.8) than the pseudo‐likelihood method (52.2). However, these methods did not give significantly different estimates (Wilcoxon signed‐rank test, *P* = 0.67) for finite populations. Effective population sizes obtained when considering a ten‐generation span were twice as large as results with a four‐generation span (Fig. 3). Given the small sizes observed, the number of generations generally had a low influence on estimates of effective population size.

**Table 1:** Characterization of the polymorphic microsatellite loci observed in *Heterodera schachtii* in four beaches and during two sampling session: number of alleles (Nall), number of individuals typed (*n*), and expected heterozygosity (He)

	Granville Nord					Granville Sud					Saint Léonard					Montfarville				
Locus		*n*		He			*n*		He			*n*		He			*n*		He	
	Nall	2012	2013	2012	2013	Nall	2012	2013	2012	2013	Nall	2012	2013	2012	2013	Nall	2012	2013	2012	2013
Hs55	3	241	280	0.470	0.465	3	189	244	0.348	0.351	3	166	154	0.423	0.415	1	164	172	0.000	0.000
Hs68	4	240	280	0.481	0.476	4	189	242	0.485	0.509	5	165	156	0.492	0.502	6	140	141	0.539	0.560
Hs33	4	240	269	0.168	0.136	4	184	229	0.135	0.109	1	157	144	0.000	0.000	1	101	94	0.000	0.000
Hs36	2	198	211	0.269	0.198	2	141	167	0.299	0.295	2	146	117	0.130	0.068	2	151	147	0.204	0.134
Hs56	3	241	274	0.038	0.078	2	189	242	0.132	0.111	2	166	155	0.245	0.210	2	165	164	0.453	0.451
Hs84	7	239	270	0.452	0.473	5	181	240	0.474	0.468	5	165	154	0.410	0.347	6	151	161	0.635	0.582
Hs111	5	241	281	0.519	0.555	6	189	240	0.525	0.514	5	166	156	0.458	0.430	3	165	172	0.543	0.531
Hs114	5	241	280	0.578	0.582	6	189	242	0.573	0.542	5	166	156	0.403	0.428	4	165	171	0.594	0.538
Mean	4.1	235.1	268.1	0.372	0.370	4	181.4	230.8	0.371	0.362	3.5	162.1	149.0	0.32	0.300	3.1	150.3	152.8	0.371	0.350

**Table 2:** Characteristic of *Heterodera schachtii* populations: number of individuals typed (n), allelic richness (Ar), expected heterozygosity (He), and deviation from HW proportions (*F*_IS_). Significant deviations from HW proportions are presented in bold characters

Site	Population	Code	*n*		Ar		He		*F*_IS_	
			2012	2013	2012	2013	2012	2013	2012	2013
Granville Nord	1	Fra.71N.P1.4	25	18	2.09	2.04	0.409	0.390	0.085	0.124
	2	Fra.71N.P2.1	25	23	1.78	1.96	0.324	0.361	−**0.162**	**0.143**
	3	Fra.71N.P2.2	25	16	1.93	1.94	0.361	0.346	0.056	**0.284**
	4	Fra.71N.P2.3	25	30	2.06	2.06	0.390	0.384	0.021	0.094
	5	Fra.71N.P2.4	23	27	1.90	2.07	0.358	0.396	0.021	**0.137**
	6	Fra.71N.P2.5	22	27	2.08	2.04	0.412	0.380	−0.04	**0.189**
	7	Fra.71N.P2.8	22	35	2.07	2.05	0.371	0.380	0.071	**0.151**
	8	Fra.71N.P3.7	21	35	2.01	2.00	0.398	0.388	0.051	**0.192**
	9	Fra.71N.P4.1	27	38	1.89	1.83	0.331	0.306	0.103	**0.170**
	10	Fra.71N.P5.2	26	32	1.92	1.93	0.361	0.361	**0.162**	**0.171**
Granville Sud	11	Fra.7_1.P3.7	19	19	2.02	2.02	0.421	0.415	**0.144**	0.129
	12	Fra.7_1.P4.1	20	22	1.85	1.89	0.339	0.333	0.136	**0.185**
	13	Fra.7_1.P5.1	22	25	2.29	2.06	0.445	0.407	0.101	**0.222**
	14	Fra.7_1.P5.2	25	25	1.94	1.86	0.341	0.312	**0.145**	0.092
	15	Fra.7_1.P5.3	25	32	1.79	1.80	0.294	0.312	0.030	−0.076
	16	Fra.7_1.P5.4	20	30	1.91	1.93	0.344	0.355	0.014	**0.191**
	17	Fra.7_1.P5.6	20	23	2.11	2.05	0.381	0.374	0.036	**0.281**
	18	Fra.7_1.P5.7	25	32	2.22	2.08	0.418	0.376	0.008	**0.099**
	19	Fra.7_1.P5.8	13	37	2.01	2.08	0.349	0.367	0.080	0.041
Saint Léonard	20	Fra.7_4.P1.1	21	25	2.06	1.82	0.398	0.316	−**0.243**	**0.201**
	21	Fra.7_4.P1.2	24	19	1.86	1.89	0.325	0.344	−0.054	**0.362**
	22	Fra.7_4.P1.3	13	11	1.87	1.73	0.335	0.276	0.025	0.141
	23	Fra.7_4.P1.7	24	22	1.82	1.91	0.292	0.365	−**0.218**	−0.077
	24	Fra.7_4.P1.8	24	17	1.84	1.70	0.339	0.293	0.096	0.125
	25	Fra.7_4.P1.9	19	28	1.97	1.87	0.368	0.328	0.070	0.058
	26	Fra.7_4.P2.1	29	17	1.86	1.71	0.318	0.296	0.063	0.138
	27	Fra.7_4.P3.5	12	17	1.53	1.43	0.188	0.172	−0.140	−0.112
Montfarville	28	Fra.8_4.P1.1	25	21	1.96	1.92	0.354	0.339	**0.391**	**0.199**
	29	Fra.8_4.P1.2	25	22	2.10	1.87	0.377	0.314	**0.269**	**0.294**
	30	Fra.8_4.P1.3	25	24	2.12	1.98	0.408	0.351	**−0.307**	**0.128**
	31	Fra.8_4.P1.4	22	33	2.00	1.92	0.343	0.315	**0.192**	**0.235**
	32	Fra.8_4.P1.5	28	26	2.14	2.04	0.385	0.371	0.097	**0.262**
	33	Fra.8_4.P2.7	17	24	2.04	1.96	0.375	0.374	**0.211**	**0.205**
	34	Fra.8_4.P2.9	23	22	2.08	2.12	0.345	0.368	**0.165**	**0.360**

**Table 3:** Effective population sizes of *Heterodera schachtii* when considering a seven‐generation span

Beach	Population	Plant status in 2013	Estimated effective size (95% confidence interval)	
			Moment based (Jorde and Ryman 2007)	Pseudo‐likelihood (Wang 2001)
Granville Nord	1	Alive	329 (127–626)	548 (48–∞)
	2	Alive	46 (15–94)	203 (59–∞)
	3	Alive	37 (12–77)	175 (40–∞)
	4^a^	Dead	∞ (∞–∞)	∞ (147–∞)
	5^a^	Dead	∞ (∞–∞)	∞ (145–∞)
	6	Dead	48 (19–89)	98 (35–1430)
	7	Dead	1224 (492–2283)	284 (57–∞)
	8^a^	Dead	∞ (∞–∞)	∞ (114–∞)
	9^a^	Dead	∞ (∞–∞)	∞ (125–∞)
	10^a^	Dead	∞ (∞–∞)	∞ (121–∞)
Granville Sud	11	Dead	113 (39–226)	353 (40–∞)
	12^a^	Alive	239 (78–489)	∞ (97–∞)
	13	Dead	650 (280–1171)	102 (36–1495)
	14^a^	Dead	∞ (∞–∞)	∞ (90–∞)
	15	Dead	190 (57–401)	975 (92–∞)
	16^a^	Dead	∞ (∞–∞)	553 (59–∞)
	17	Dead	111 (44–207)	444 (59–∞)
	18^a^	Dead	∞ (∞–∞)	417 (60–∞)
	19^a^	Dead	∞ (∞–∞)	∞ (112–∞)
Saint Léonard	20	Dead	39 (15–74)	43 (19–146)
	21	Alive	162 (59–315)	175 (36–∞)
	22	Alive	256 (70–560)	67 (13–∞)
	23	Alive	65 (19–136)	136 (29–∞)
	24	Alive	21 (6–44)	27 (10–90)
	25^a^	Dead	∞ (∞–∞)	∞ (100–∞)
	26	Alive	373 (129–743)	124 (29–∞)
	27^a^	Alive	∞ (∞–∞)	264 (12–∞)
Montfarville	28^a^	Alive	∞ (∞–∞)	1527 (56–∞)
	29	Alive	40 (16–75)	44 (20–139)
	30	Alive	51 (21–94)	43 (22–108)
	31	Alive	140 (51–273)	76 (27–628)
	32	Alive	342 (132–651)	90 (29–6905)
	33	Dead	133 (49–259)	176 (32–∞)
	34^a^	Dead	160 345 (58 793–311 870)	∞ (73–∞)

a indicate populations with an infinite estimate of effective size.

### Impact of nonrandom forces

The exact homogeneity tests detected significant changes in allele frequencies between the two sampling sessions in nine populations. Of these, only population two exhibited a significant action of nonrandom forces such as selection or migration (Waples test, *P* < 0.05, Table S1).

## Discussion

The aim of this study was to estimate the effective population size of *H. schachtii* in wild ecosystems. Despite the occurrence of 14 populations showing infinite *N*_*e*_ estimates for at least one of the estimators used, the 20 other effective population sizes ranged between 20 and 1300 individuals, with most of them having an *N*_*e*_ close to 80–90 individuals.

### Requirements and reliability of effective population size estimates by temporal sampling

Most of the single‐sample estimators are biased in cases of strong assortative mating or population substructuring (Zhdanova and Pudovkin 2008; Waples and Do 2010; Holleley et al. 2014). We therefore used a temporal approach to estimate effective population sizes. However, temporal methods also have underlying assumptions that can greatly affect the reliability of effective size estimates if they are violated. First, they require that temporal samples be separated by several generations. As the generation time of *H. schachtii* in the wild is poorly documented, it was necessary to approximate this parameter using data from field nematode populations (Subbotin et al. 2010) and previous experiments (Kakaire et al. 2012). We determined that seven generations, plus or minus three, elapsed between samples. Second, temporal methods assume discrete generations, which did not necessarily hold in our case study. However, the determined number of generations between sampling sessions was high enough to reduce the influence of overlapping generations as shown by Waples and Yokota (2007), although there is likely a small upward bias for populations with only four or five generations.

In the case of *H. schachtii*, however, an additional difficulty is that cysts sampled in 2013 may potentially come from the same generation as cysts sampled in 2012, because cysts can survive in the soil for many years in the case of unfavorable environmental conditions. Therefore, few generations may have elapsed between the two sampling sessions, leading to insufficient genetic variation for estimating effective population size. This may explain the frequent occurrence of infinite population effective sizes. In fact, 79% of the host plants tagged and characterized by infinite nematode population size died between the two sampling sessions (Table 3). However, 40% of finite‐size populations also were associated with dead host plants, suggesting that plant death does not lead directly to infinite effective population sizes. This could be explained by the survival of underground plant parts or the recent death of the host plant. Infinite effective population sizes could also simply reflect populations with huge effective sizes (*N*_*e*_ > 35000, see the Methods and Results sections above). Variation of effective population size between populations could correspond to source‐sink dynamics (Manier and Arnold 2005; Barson et al. 2009). Such metapopulation models, however, are unlikely to apply to species which, like *H. schachtii*, do not disperse between host plants (C. Gracianne, P.‐L. Jan, S. Fournet, E. Olivier, J.‐F. Arnaud, C. Porte, S. Bardou‐Valette, M.‐C. Denis and E. J. Petit, unpublished data).

**Figure 4:**
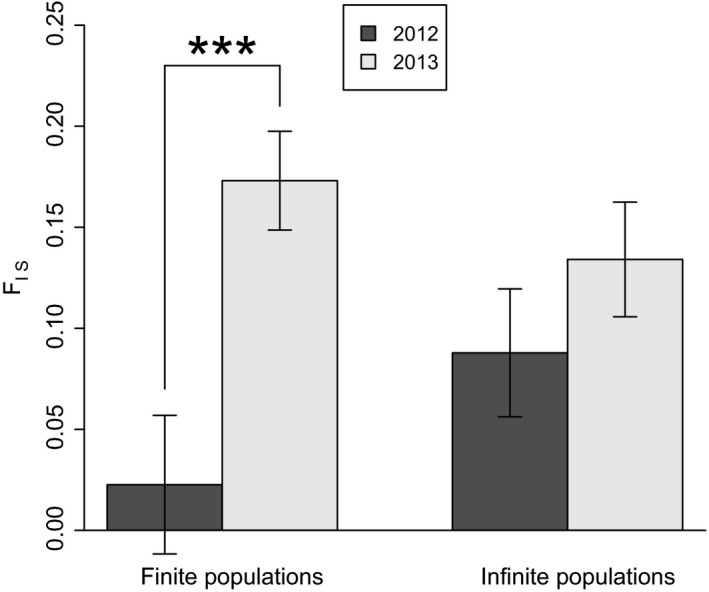
Variation of mean *F*_IS_ of finite and infinite populations over time. Error bars represent standard error. ***: *P* < 0.001.

The analysis of temporal variation in mean multilocus *F*_*IS*_ estimates can give further information on the likelihood that new generations were found in the 2013 samples. The life cycle of *H. schachtii* and the low dispersal ability of larvae result in inbreeding and/or substructuring (Wahlund effect), which leads to an increase of heterozygote deficiencies over time (Plantard and Porte 2004; Montarry et al. 2015). However, this increase in heterozygote deficiencies is possible only in populations that produce new generations. As shown in Fig. 4, an increase was indeed observed for populations with finite effective sizes, but not in populations with infinite effective sizes (paired permutation test; 10 000 permutations; *P* < 0.001 and *P* = 0.12, respectively). Furthermore, the number of cysts observed in our samples (corrected by the proportion of *H. schachtii* cysts relative to other species) was remarkably similar between finite and infinite populations (permutation test; 10 000 permutations; *P* = 0.60), with a mean number of cysts 158.2 ± 19.8 (SEM) and 175 ± 23.8, respectively, refuting the hypothesis that infinite population size results from huge census sizes.

In addition, infinite estimate of effective population size also may be caused by an overestimation of the number of generations between samples, which reduces the effect of genetic drift. However, all infinite *N*_e_ estimates remained infinite when we decreased the number of generations from ten to four. Overall, our findings suggest that infinite population sizes correspond to nematode populations lacking significant temporal variations in allele frequencies, probably because new nematode generations were not produced between the two sampling sessions.

Temporal methods assume no significant influence of other micro‐evolutionary forces, such as migration or selection. The relevance of this assumption in our dataset was tested by applying a generalized test developed by Waples (1989): only population two exhibited a significant impact of nonrandom forces. The effective population size estimated by the pseudo‐likelihood method, used to compute this test, was much higher than that of the moment‐based method, for which the Waples test was nonsignificant (data not shown). Thus, it is possible that this population was not affected by nonrandom evolutionary forces, and *N*_e_ was overestimated for this population. Therefore, we considered that our populations were not affected strongly by migration or selection during the one‐year interval between the two samples and that the assumptions of the temporal method held for the finite‐size populations, thereby giving reliable estimates.

Population density in soil samples was around 40 cysts of *H. schachtii* per 100 g of soil. Assuming that each cyst contains between 500 and 600 eggs (Subbotin et al. 2010), the census size *N* of the populations we sampled was, at least, 10 000 individuals, while both estimators yielded effective populations sizes that were less than 1300 individuals, with a majority of effective population sizes around 85 individuals. Thus, the *N*_*e*_ */N* ratio was less than 1% for most populations, which is extremely low. Low effective sizes of wild populations are common, but the *N*_*e*_/*N* ratios in these populations are generally closer to 10% (Frankham 1995; Palstra and Ruzzante 2008). It is unlikely that we underestimated the effective population sizes because both *N*_e_ estimators gave very similar results. The pseudo‐likelihood estimator is more reliable when considering precision and bias (Gilbert and Whitlock 2015), but can be slightly upwardly biased in specific cases, whereas the moment‐based estimator has been demonstrated to be essentially unbiased (Jorde and Ryman 2007). Our low estimates of effective population sizes are thus conservative.

### Insights gleaned from the estimation of effective population sizes for cyst nematodes

The effective population size is affected by many population features, three of which may dramatically influence effective sizes in *H. schachtii*. First, the factor with the highest impact on effective population size is the variation in population size through time (Frankham 1995; Charlesworth 2009). In the case of *H. schachtii*, the death of the host and the recolonization of a patch by host plants may eventually lead to variation in population census sizes. In Normandy, *B. maritima* lives seven to nine years on average (Hautekèete et al. 2002), and a significant rate of host death is unexpected for a one‐year period. However, the high number of dead plants observed in the second sampling session supports the hypothesis of host instability. The death of these plants can be attributed to disturbance events such as storms (De Cauwer et al. 2012), but another possibility is that *B. maritima* populations contain numerous annual individuals along with biennial and perennial individuals (Biancardi et al. 2012). Moreover, the distribution of effective size appears to fit a log‐normal distribution (Fig. 2), which is common in populations living in a fluctuating environment (Lewontin and Cohen 1969). These facts suggest that *H*. *schachtii* lives in an environment in which local conditions often fluctuate, directly affecting the census size of the population, and possibly explaining the low effective size of their populations.

Second, low effective population sizes also can be explained by certain life‐history traits in *H. schachtii*. Its populations are characterized by high levels of inbreeding (Plantard and Porte 2004), which increases the correlation between maternal and paternal alleles, and thus increases the impact of genetic drift and thus reduces effective population sizes (Charlesworth 2009). Third, the sex ratio in *H. schachtii* can be extremely unbalanced because sex determination is environmental and depends on the quality of the host, resulting in a male/female ratio that can vary from 0.40 when conditions are optimal to 29.0 on resistant hosts (Grundler et al. 1984; Müller 1985; Grundler and Böckenhoff 1997). The wild sea beet has been long recognized as a resistant host plant species for *H. schachtii* (see Panella and Lewellen 2007). Wild populations of *H. schachtii* may thus have very unbalanced sex‐ratios, as well as high variance among individuals in reproductive success. Unequal sex‐ratios and variance in offspring number are two major factors that reduce *N*_e_ below the census size of the population (Charlesworth 2009). However, the actual resistance of the wild sea beets sampled in this study is unknown, and its possible impact on cyst nematode sex‐ratios requires further investigation.

### Implications of wild effective population size for the control of cyst nematode populations

This study demonstrated that wild populations of *H. schachtii* have effective population sizes of around 85 individuals with a *N*_*e*_ */N* ratio less than 1%. This is consistent with *N*_*e*_ estimations of another cyst nematode, *Globodera pallida*, which also yields a *N*_*e*_ */N* ratio of less than 1% at the host scale in laboratory conditions (Montarry, personal communication). For comparison, estimates of the effective population size of the free‐living nematode *Caenorhabditis elegans* range (i) from 200 to 9600 individuals with AFLP markers (Barrière and Félix 2005); (ii) from 50 to 10 200 with microsatellite markers (Sivasundar and Hey 2005); and (iii) around 80 000 with full‐length sequences (Cutter 2006). The census sizes of most *C. elegans* populations are of the order of 10 000 individuals or less (Félix and Duveau 2012). The effective size of an animal parasitic nematode, *Trichostrongylus axei*, is estimated to be of the order of 10 million at the metapopulation scale (Archie and Ezenwa 2011). Comparison of these data with ours have to be made with caution, because we only estimated effective size at the host scale; however, macroparasite models predict an even lower *N*_*e*_ */N* ratio when considering the whole metapopulation, mainly due to the subdivision of breeders between hosts (Criscione and Blouin 2005).

Thus, effective population sizes found in *H. schachtii* may be considered as surprisingly low compared with other nematodes. However, these results are consistent with the work of Barrett et al. (2008), which predicted a low effective population size for macroparasite populations subject to inbreeding, restricted dispersal, and development on short‐lived hosts like *H. schachtii* (*T. axei*, however, maintains very high rates of gene flow). Low effective sizes have been observed in other plant pathogens such as fungi (Bayon et al. 2009) and viruses (Sentandreu et al. 2006; Fabre et al. 2012), although not in all cases (Zhan and McDonald 2004; Gurung et al. 2013; and see McDonald and Linde 2002 for a review).

Low effective population sizes imply that genetic drift strongly affects wild populations of *H. schachtii*. Current knowledge on nematode mutation (Baer et al. 2007) and *H. schachtii* migration (C. Gracianne, P.‐L. Jan, S. Fournet, E. Olivier, J.‐F. Arnaud, C. Porte, S. Bardou‐Valette, M.‐C. Denis and E. J. Petit, unpublished data) rates suggest that these two evolutionary forces are too weak to enhance the evolutionary potential of these populations at the host scale. Finally, this calls into question the capacity of this nematode to adapt to changing environments unless selection intensity is strong (Charlesworth 2009). This makes it unlikely that wild populations could serve as a source of virulent cyst nematodes.

If actual effective field population sizes of *H. schachtii* are similar to the ones estimated in this study, then the genetic resistance used in sugar beet fields could remain effective for a long time (Van der Putten et al. 2006). However, populations of *H. schachtii* on crops probably differ from wild populations (Porte et al. 1999; Plantard and Porte 2004). Plowing and other agricultural practices could increase their effective size by mixing populations. Higher nematode densities can be expected in field populations as compared with wild populations because of the planting of crops favorable to nematode development, such as alternative *H. schachtii* hosts (*e.g*., oil seed rape) or tolerant beet varieties (S. Fournet and C. Porte, unpublished data). Given that our results have shown that *H. schachtii* populations are naturally vulnerable to genetic drift without anthropogenic influence, clever use of this feature could prevent the parasite from overcoming plant resistance. Crop rotation is an efficient way to modify population census size of *H. schachtii* through time, by introducing large variation in host availability, similar to that experienced in wild populations. This method was once considered as difficult to implement for control of *H. schachtii*: this nematode is a generalist able to parasitize most *Brassicaceae* species. Crop and weed species that can be used to reduce the census size of *H. schachtii* are now known, leading to the development of nematicidal intercrops (Curto 2008; Meinecke and Westphal 2014). Another way to influence effective population size is to enhance the effect of host on the sex ratio of *H. schachtii* (Grundler et al. 1984; Müller 1985) through resistance genes or crops used for fallow. Agricultural management that promotes small effective population sizes in the fields would be greatly beneficial for the sustainable use of plant resistance to manage *H. schachtii* populations.

Further comparative studies are needed to identify which factors are responsible for the low effective size of wild *H. schachtii* populations. For instance, fine‐scaled substructuring or demographic fluctuations must be further documented to improve our understanding of the evolutionary dynamics and history of *H. schachtii*. As demonstrated here, low effective population sizes give insights regarding potential control methods for *H. schachtii* in sugar beet fields to preserve the effectiveness of resistant varieties over the long term. We recommend future studies on the estimation of effective population sizes of crop pests, in both natural environments and agrosystems, to identify the factors that best control crop pathogen populations.

## Data archiving statement

Data for this study are available from the Dryad Digital Repository: http://dx.doi.org/10.5061/dryad.65bd3.

## Supporting information


**Figure S1.** Generation time of *Heterodera schachtii* as a function of temperature.

**Figure S2.** Mean Temperature (bar plots) and maximum number of generations of *Heterodera schachtii* produced every month between the two sampling sessions (lines).

**Table S1.** Waples generalized test results for populations with significant temporal changes in allelic frequencies (exact homogeneity test).
