# Supplementary material for: Temporal sampling helps unravel the genetic structure of naturally occurring populations of a phytoparasitic nematode. 1. Insights from the estimation of effective population sizes
Source: Evol Appl. 2016 Feb 11;9(3):489–501. doi: 10.1111/eva.12352 (PMC4778111; doi:10.1111/eva.12352)
Supplement: Supplementary file 1 — Figure S1. Generation time of Heterodera schachtii as a function of temperature. [file EVA-9-489-s001.docx]

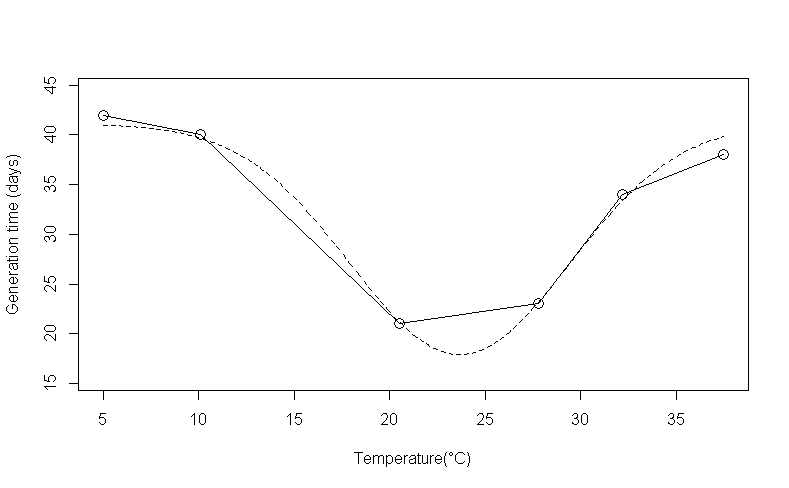
**Figure S1 Generation time of *Heterodera schachtii* as a function of temperature.** The solid line corresponds to the data from Kakaire et al. (2012) and the dotted line to the nonlinear regression model.

Kakaire, S., I. G. Grove, and P. P. Haydock. 2012. Effect of Temperature on the Life Cycle of *Heterodera Schachtii* Infecting Oilseed Rape (Brassica Napus L.). *Nematology* 14 (7): 855–67.
