# Supplementary material for: Temporal sampling helps unravel the genetic structure of naturally occurring populations of a phytoparasitic nematode. 1. Insights from the estimation of effective population sizes
Source: Evol Appl. 2016 Feb 11;9(3):489–501. doi: 10.1111/eva.12352 (PMC4778111; doi:10.1111/eva.12352)

**Figure S2 Mean Temperature (bar plots) and maximum number of generations of *Heterodera schachtii* produced every month between the two sampling sessions (lines).** Black: Montfarville; Grey: Granville Nord, Granville Sud and Saint-Léonard.


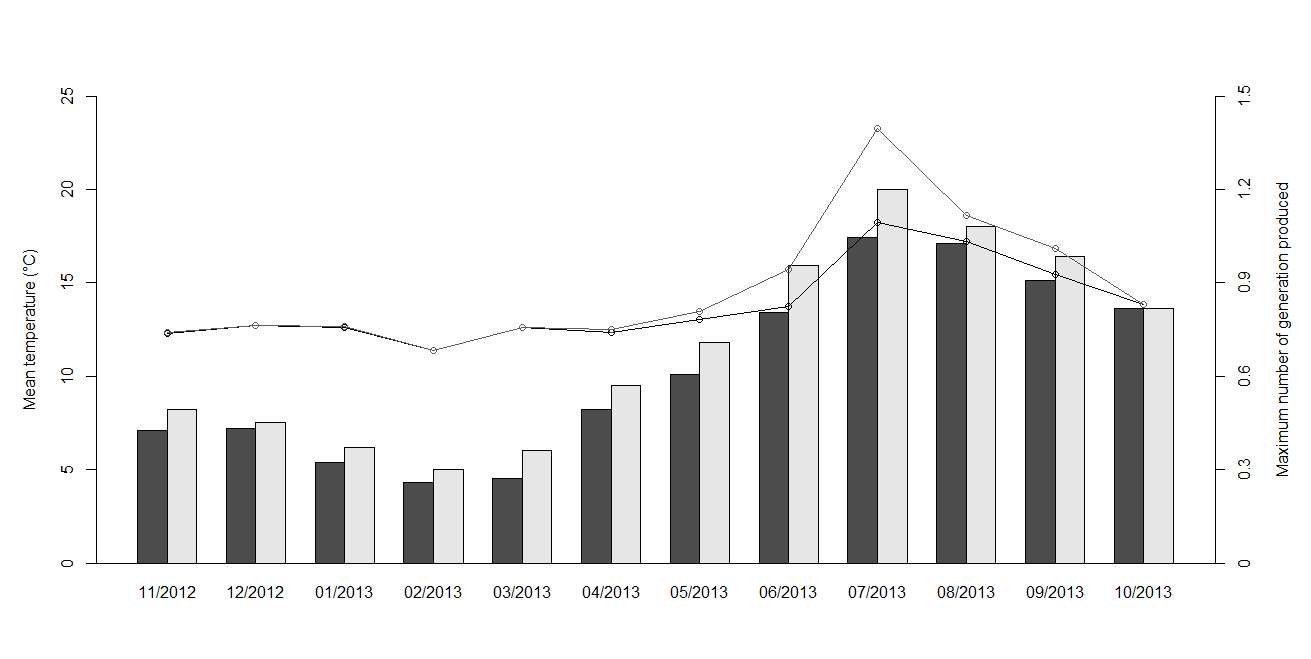

Supplement: Supplementary file 2 — Figure S2. Mean Temperature (bar plots) and maximum number of generations of Heterodera schachtii produced every month between the two sampling sessions (lines). [file EVA-9-489-s002.docx]
