# Supplementary material for: Temporal sampling helps unravel the genetic structure of naturally occurring populations of a phytoparasitic nematode. 1. Insights from the estimation of effective population sizes
Source: Evol Appl. 2016 Feb 11;9(3):489–501. doi: 10.1111/eva.12352 (PMC4778111; doi:10.1111/eva.12352)
Supplement: Supplementary file 3 — Table S1. Waples generalized test results for populations with significant temporal changes in allelic frequencies (exact homogeneity test). [file EVA-9-489-s003.docx]

**Table S1.** Waples generalized test results for populations with significant temporal changes in allelic frequencies (exact homogeneity test).

| **Population** | **Effective size** | **Waples's test** |
| --- | --- | --- |
| 2 | 203 | * |
| 6 | 98 | n.s |
| 13 | 102 | n.s |
| 20 | 43 | n.s |
| 24 | 27 | n.s |
| 29 | 44 | n.s |
| 30 | 43 | n.s |
| 31 | 76 | n.s |
| 32 | 90 | n.s |
